# Efficacy and safety of nilotinib as frontline treatment in elderly (> 65 years) chronic myeloid leukemia patients outside clinical trials

**DOI:** 10.1007/s00277-023-05159-9

**Published:** 2023-04-20

**Authors:** Luigia Luciano, Roberto Latagliata, Gabriele Gugliotta, Mario Annunziata, Mario Tiribelli, Bruno Martino, Antonello Sica, Maria Rosaria Esposito, Monica Bocchia, Sara Galimberti, Federica Sorà, Francesco Albano, Raffaele Palmieri, Patrizia Pregno, Matteo Dragani, Maria Iovine, Simona Sica, Alessandra Iurlo, Fausto Castagnetti, Gianantonio Rosti, Massimo Breccia

**Affiliations:** 1 Hematology Unit, Federico II” University of Naples, Naples, Italy; 2 Department of Cellular Biotechnologies and Hematology, University “La Sapienza” of Rome, Rome, Italy; 3 Institute of Hematology “L. and A. Seràgnoli”, Department of Experimental, Diagnostic and Specialty Medicine, “S. Orsola-Malpighi” University Hospital, University of Bologna, Bologna, Italy; 4 Hematology Unit, Cardarelli Hospital, Naples, Italy; 5 Division of Hematology and BMT, Azienda Ospedaliero - Universitaria Di Udine, Udine, Italy; 6 Hematology Unit, Bianchi Melacrino Morelli Hospital, Reggio Calabria, Italy; 7 Hematologyunit, “L Vanvitelli” University of Campania, Naples, Italy; 8 Hematologyunit, Ascalesi Hospital, Naples, Italy; 9 Hematology Unit, Azienda Ospedaliera Universitaria Senese and University of Siena, Siena, Italy; 10 Department of Clinical and Experimental Medicine, Section of Hematology, University of Pisa, Pisa, Italy; 11 Department of Diagnostic Imaging, Oncological Radiotherapy and Hematology, A. Gemelli, IRCCS University Hospital Foundation, Rome, Italy; 12 Hematology and Transplants Unit, University of Bari, Bari, Italy; 13 Hematology Unit,“S. Giuseppe Moscati” Hospital, Avellino, Italy; 14 Hematology Unit, Azienda Ospedaliero Universitaria Città Della Salute E Della Scienza, Turin, Italy; 15 Hematology, San Luigi Gonzaga Hospital, Orbassano, Italy; 16 Hematology Unit, AO “S. Anna E S. Sebastiano”, Caserta, Italy; 17 Oncohematology Division, IRCCS Ca’ Granda - Maggiore Policlinico Hospital Foundation, University of Milan, Milan, Italy

**Keywords:** Chronic myeloid leukemia, Elderly, Nilotinib, Efficacy and safety

## Abstract

Here, we report real-world evidence on the safety and efficacy of nilotinib as a first-line treatment in elderly patients with chronic phase CML, treated in 18 Italian centers. Sixty patients aged > 65 years (median age 72 years (65–84)) were reported: 13 patients were older than 75 years. Comorbidities were recorded at baseline in 56/60 patients. At 3 months of treatment, all patients obtained complete hematological response (CHR), 43 (71.6%) an early molecular response (EMR), while 47 (78%) reached a complete cytogenetic response (CCyR). At last follow-up, 63.4% of patients still had a deep molecular response (MR4 or better), 21.6% reached MR3 as best response and 11.6% persisted without MR. Most patients (85%) started the treatment at the standard dose (300 mg BID), maintained at 3 months in 80% of patients and at 6 months in 89% of them. At the last median follow-up of 46.3 months, 15 patients discontinued definitively the treatment (8 due to side effects, 4 died for unrelated CML causes, 1 for failure, 2 were lost to follow-up). One patient entered in treatment-free remission. As to safety, 6 patients (10%) experienced cardiovascular events after a median time of 20.9 months from the start. Our data showed that nilotinib could be, as first-line treatment, effective and relatively safe even in elderly CML patients. In this setting, more data in the long term are needed about possible dose reduction to improve the tolerability, while maintaining the optimal molecular response.

## Introduction

Far less data is available on the efficacy and safety of second-generation TKIs as first-line treatment in chronic myeloid leukemia (CML) of the elderly. This category of patients is usually not enrolledin sponsored clinical trials due to inclusion criteria which exclude the majority of patients with specific comorbidities, much more frequent with ageing. In real-life, the concomitance of comorbidities and other specific medications might prevent optimal management and treatment to possible increased specific TKI-related toxicity, especially with frontline second-generation TKIs. Before TKI era, increased age represents an adverse prognostic factor and was included in the two most used prognostic scores for CML, the Sokal score and EURO score, among parameters significantly impacting on outcome. Consequently, a higher proportion of older patients are at intermediate or high risk [1]. Indeed, in the TKI era, the EUTOS score did not identify age as a risk variable and, more recently, the ELTS score which differentiates the probabilities of dying of CML better than the previous scores includes age but with a less important role. After the introduction of TKI treatment in clinical practice, elderly CML patients have been treated with imatinib: this strategy changed completely the outcome and increased the survival even in the older subset [2, 3]. Over time, some real-life trials have been reported result of effectiveness and safety of second-generation TKIs in the elderly subset too [4]. Moreover, a sub-analysis of the ENEST1st study showed that age did not have a relevant impact on the deep molecular response rate associated with frontline nilotinib treatment in newly diagnosed CML patients and showed no differences if compared to younger population even for the eligibility to attempt treatment discontinuation (TFR) [5]. A recent update of ENEST Freedom trial evaluated the efficacy and safety of TFR after upfront treatment with NIL in older (≥ 65 years at study entry) vs younger (< 65 years). The results showed that a lower proportion of pts ≥ 65 years who achieved MR4.5 following upfront treatment with NIL for ≥ 2 years remained in MMR/MR4.5 after NIL discontinuation compared with pts < 65 years [6]. Therefore, it is particularly important to evolve age-related policies of CML treatment, to ensure adequate treatment also for this age group, always considering side effects and comorbidities.

We report here a real-life multicenter experience with nilotinib as first-line treatment in a cohort of elderly CML patients in CP over the age of 65 years, treated in 17 Italian centers in order to evaluate the drug’s efficacy and safety in this setting of patients.

## Patients and methods

We retrospectively collected data related to 60 elderly patients with CP-CML treated with nilotinib as first-line treatment to report efficacy and safety outside of clinical trials, except 5 patients enrolled in Italian clinical trials (8%). Each participating center provided the required data, after obtaining an informed consent according to ethics committee. No patient was excluded and even patients who discontinued for toxicity have been considered. All the 60 Ph + and/or BCR-ABL1 + CML pts were in early CP. Diagnostic criteria were based on the ELN criteria and the risk scores were defined following the Sokal and ELTS scores [7, 8]. Complete cytogenetic response (CCyR), MMR (*BCR-ABL1*^IS^ < 0.1%), and deep molecular responses (DMR) (MR4.0 = *BCR-ABL1*^IS^ < 0.01%; MR4.5 = *BCR-ABL1*^IS^ < 0.0032%; MR5.0 = *BCR-ABL1*^IS^ < 0.001) were defined according to the standardized criteria [9] and according to the International Scale (IS). The data have been collected between 2008 and 2017; half of the patients were enrolled between 2012 and 2014. The median follow-up was of 46.3 months. The rate and severity of hematologic and non-hematologic AEs were assessed according to the CTCAE 4.0 scale.

Molecular analyses were performed by RQ-PCR in certified laboratories and responses were defined according to the ELN 2013 recommendations [10]. Molecular monitoring was performed every 3 months. OS was calculated from the date of diagnosis until death at any time for any reason; PFS was calculated from the date of start of treatment until progression to accelerated phase (AP) or blast phase (BP) at any time; event-free survival (EFS) was calculated from the date of start treatment until death, progression to AP or BP, failure on nilotinib, or treatment discontinuation for any cause. Resistance to nilotinib was retrospectively defined according to the current ELN criteria [9]; intolerance was considered as a condition that due to the severity and/or receptiveness of one or more drug-related side effects led to treatment discontinuation.

## Results

Sixty elderly patients with CP-CML were collected: the median age was 72 years, with a male/female ratio of 30/30. Thirteen patients were older than 75 years. All patients were confirmed Philadelphia positive at diagnosis by chromosome banding analysis. Qualitative RT-PCR revealed the presence of b3a2 transcript in 37 patients, 5 patients co-expressed both transcripts, and 1 patient showed the e1a2 transcript while the remaining showed the b2a2 transcript (Table 1). The SOKAL score was high in 18 patients (30%), intermediate in 36 patients (60%), and low in only 6 patients (10%).

**Table 1:** Patient characteristics

Patients, *n*	60
Age, years, median (range)	72 (61–84)
Sex, *n* (M/F)	30/30
Sokal risk, *n* (%)	
• Low • Intermediate • High	6 (10%) 36 (60%) 18 (30%)
ELTS score, *n* (%)	
• Low • Int • High	12 (20%) 38 (63.3%) 10 (16.6%)
CCI, median (range)	2 (0–5)
ECOG, median (range)	0 (0–2)
Variant translocations, *n* (%)	3 (5%)
Transcript type, *n* (%)	
• b3a2 • b2a2 • both • e1a2	37 (62%) 17 (28%) 5 (8%) 1 (2%)
Comorbidity at diagnosis, *n*	56
Cardiovascular, *n* (%)	
✓ Hypertension ✓ Ischemic heart disease ✓ Atrial fibrillation ✓ Carotid stenosis	35 (62%) 3 (5%) 3 (5%) 2 (3%)
Methabolic disorders, *n* (%)	
✓ Diabetes ✓ Hypercholesterolemia	7 (12%) 9 (16%)
Other, *n* (%)	
✓ Hypothyroidism ✓ Autoimmune disease ✓ Previous cancer	4 (7%) 5 (9%) 8 (14%)

Baseline comorbidities were recorded in 56 patients (93%) (Table 1): the Charlson Comorbidity Index (CCI) calculated at baseline was prevalently between 0 and 3 for most patients except for 10 who showed a CCI of 4–5. Concomitant drugs were recorded in 51 patients. The ECOG score was 0 in most patients (Table 1).

Fifty-two patients started treatment at the standard dose (300 mg BID), while 6 patients received 450 mg per day and 2 patients started at 300 mg QD. These patients who started on low dose were all aged more than 75 years and presented severe comorbidities at baseline.

At 3 months of treatment, all patients obtained a complete hematological response (CHR). Forty-seven patients (78.3%) showed a complete cytogenetic response (CCyR), 2 showed a partial cytogenetic response (PCyR), and 3 did not obtain any response. In 8 patients, cytogenetic was not evaluable.

An early molecular response (EMR) was reached by 43 patients (71.6%); in 10 (16.6%) patients, the response was not evaluable, while 3 patients failed to reach any molecular response. Table 2 shows the evolution of the molecular response at 6, 12, and 24 months: 55.1%, 75.5%, and 77.3% of patients respectively obtained a MR3 or deep MR (MR4, MR4.5, MR5). At last follow-up, a deep molecular response (MR4 or better) was recorded in 63.4% of patients, while 21.6% reached a MR3 as best response and 11.6% failed to achieve any MR (Table 3). During the 24 months of follow-up, at 3 months, 82% of patients maintained the standard dose of 300 mg BID, and 5 out of 6 patients who started with 450 mg per day maintained the same dose while 1 patient switched to imatinib for intolerance. Five patients (8%) reduced the dose due to hematological toxicities; 2 patients discontinued due to non-hematological toxicity (1 for gastrointestinal toxicity, 1 for uncontrolled diabetes). At 6 months, 89% of patients maintained the standard dose, while 2 patients reduced the dose, one for anemia while was in MR3, one patient for personal choice in deep molecular response, 2 patients who started with 450 mg per day switched to imatinib for non-hematological toxicity, one patient increased the dose to 400 mg BID for resistance due to the presence of E255K mutation. At 12 months, 42/45 patients (93%) continued the drug at standard dose, while at 18 months 41 patients remained in treatment at standard dose, one patient reduced the dose of nilotinib for hematological toxicity while in deep molecular response, one discontinued nilotinib temporarily for hematological toxicity (leucopenia) and for arrhythmia for 1 month. At 24 months, 38 patients continued at standard dose, none of the patients reduced the dose at this time point, whereas one patient discontinued the treatment for pleural effusion. At the last median follow-up of 49.5 months, 8 patients discontinued definitively the treatment due to side effects and switched to another TKI. All these patients started nilotinib treatment at standard dose and they did not reduce the dose before discontinuation. Four patients died for unrelated CML causes, and 2 patients were lost to follow-up, while 14 patients continue the therapy at low dose and 33 patients at standard dose and only 1 patient starting at 450 mg per day maintained the same dose, while the other 4 patients reduced dose to 300 mg/die because of in MMR or DMR. Interestingly, one patient attempted the TFR and he is in continuous deep MR after 2 years (Table 3).

**Table 2:** Molecular results according to ELN recommendation

	3 months	6 months	12 months	18 months	24 months	Last follow-up
EMR	78.4%					
No MMR	5.0%	41.6%	20.7%	14.3%	15.9%	11.6%
MR3	15.0%	30.0%	35.8%	34.7%	18.1%	21.6%
MR4	1.6%	6.7%	17.0%	10.3%	13.6%	26.6%
MR4.5	1.6%	11.7%	13.3%	24.4%	22.8%	18.4%
MR5	0	6.7%	5.6%	12.3%	22.8%	18.4%
NE	16.6%	3.3%	7.6%	4.0%	6.8%	3.4%
Discont		5.4%	7.2%	9.0%		26.7%

**Table 3:** Patient disposition at last follow-up

Patients, *n*	60
Still on nilotinib, *n*	44
600 mg < 600 mg	32 12
Discontinued nilotinib, *n*	16
✓ Adverse events ✓ Failure ✓ Treatment-free remission ✓ Progression to advance phase ✓ Dead ✓ Lost to follow-up	8 1 1 0 4 2
Median follow-up, months (range)	49.5 (4.5–118.6)

As regards safety, 14 patients showed grade 2 hematological toxicity and 26 patients experienced non-hematological side effect grades 1–3 (Table 4). Most recorded side effects were G1/G2 according to CTC scale and resolved through modulation of the dose; only 1 patient showed a G3 hepatic toxicity. Of them, 4 patients discontinued nilotinib due to toxicity. Six patients experienced cardiovascular events: the onset was after a median time of 20.9 months from the start. In detail, 1 patient experienced atrial fibrillation, 1 ischemic heart disease, 1 a left ventricular hypertrophy, 1 QT interval prolongation, 1 cardiomyopathy, and 1 a cerebral stroke. All patients had at least one cardiovascular risk before treatment (hypertension). All cardiovascular side effects were G1/G2, whereas only the cerebral stroke was G3 (Table 4). All patients discontinued the drug except two who continued at a reduced dose. Most of all other patients with cardiovascular comorbidities at diagnosis did not reduce the dose of nilotinib, except for 4; 2 patients discontinued the drug due to non-CV side effects. Overall, 8 patients discontinued the drug for intolerance.

**Table 4:** Side effects

Patients, *n*	60	Grade 1/2	Grade 3
Hematological side effects, *n* (%)	14 (23.4%)	13	1
✓ Anemia ✓ Leukopenia ✓ Thrombocytopenia	9 (15.0%) 1 (1.7%) 4 (6.7%)	9 1 1	0 0 0
Non-hematological side effects, *n* (%)	26 (43.3%)		
✓ Skin ✓ Hepatic ✓ Gastrointestinal ✓ Glucose increase ✓ Cholesterol increase ✓ Fatigue	13 (21.7%) 3 (5.0%) 3 (5.0%) 3 (5.0%) 3 (5.0%) 5 (8.3%)	11 2 1 3 3 5	2 1 2 0 0 0
Cardiovascular side effects			
Cardiovascular events,* n*	6		
✓ Risk factor at baseline ✓ Age at event, years, median (range) ✓ Time from start of nilotinib, months, median (range)	Yes (hypertension) 77.6 (70–83) 20.9 (7–55)		
Type of cardiovascular event			
✓ Atrial fibrillation ✓ QT prolongation ✓ Ischemic heart disease ✓ Cardiomyopathy ✓ Ventricular hypertrophy ✓ Ischemic cerebral stroke	1 1 1 1 1 1	0 0 1 1 1 1	1 1 0 0 0 0

**Fig. 1:**
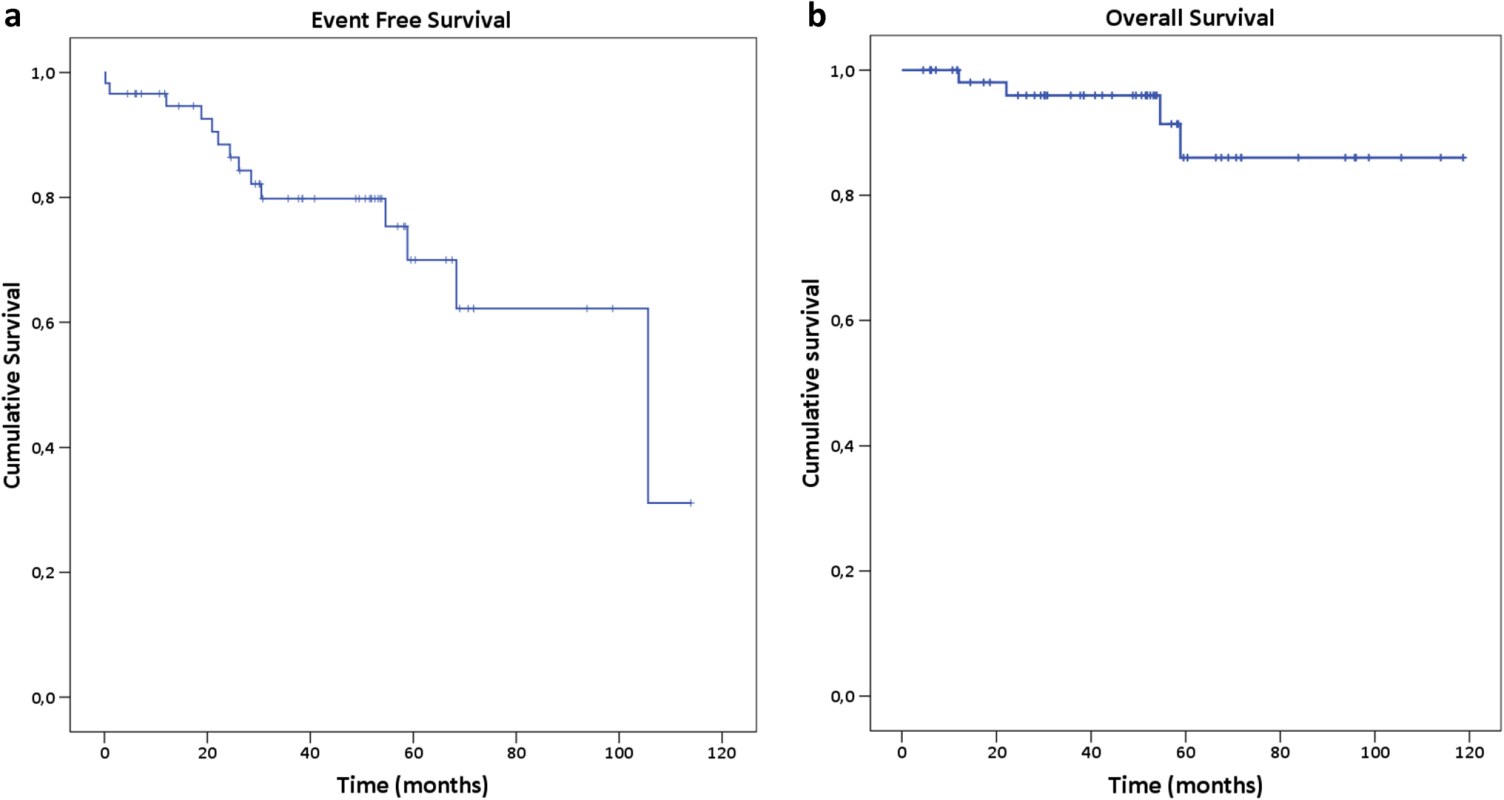
Event-free survival (EFS) and overall survival (OS)

Event-free survival (EFS) and overall survival (OS) are reported in Fig. 1 with an estimated median survival of 105.6 months (95% CI: 52.6–158.7) in EFS curve and a median not reached in OS. Median time of observation was 38.5 and 49.5 for EFS and OS, respectively. In the EFS curve, a total of 14 events defined as death for any cause, toxicity, resistance, and intolerance were observed, while only 4 events were observed in OS.

## Discussion

The role of age as prognostic factor in CML patients has changed from the pre‐TKI period to the current phase of treatment with TKI. The approval of first-generation (imatinib) and second-generation (nilotinib and dasatinib) TKIs for first-line treatment of CML allowed the possibility to treat even the elderly. Multiple studies on imatinib have shown that old age does not affect the rates of response to treatment and overall survival [3, 11–14]. In TKI-treated elderly patients, the rates of complete cytogenetic and major molecular responses were comparable to those of adult patients (30–59 years old) and even higher than those in young adults (18–29 years old), with significantly lower probability of transformation to advanced phases. However, the overall survival of elderly patients was inferior due to deaths unrelated to CML [15].

The use of second-generation TKIs (dasatinib, nilotinib, and bosutinib) in first line has shown a significantly higher proportion of patients to have optimal response at the 3-month milestone, which was recognized as an important prognostic factor and may increase the proportion of patients with deep molecular responses, who may potentially enter treatment-free remission (TFR) [16]. They are also all equally effective when comparing the response data of older patients with younger CML, in both first and second lines. Data from the BELA, ENESTnd, and DASISION studies all provide clear evidence that age is not a factor that predicts lower response rates [16–20].

The ENESTnd 10-year update analysis shows higher rates of MMR and MR4.5 with nilotinib and higher rate of sustained DMR, suggesting that nilotinib treatment is associated with long-term benefit, including the possibility of attempting TFR in general population, also in elderly patients. The overall survival and progression-free survival are similar in all 3 arms in general population but lower in the older patient subsets, result to be interpreted with caution due to the small sample size, as authors suggest [16]. Similarly, in second line, elderly patients treated with nilotinib demonstrated not only similar efficacy but also same rate of adverse events, discontinuation, and dose reduction of youngers [21].

Thus, second-generation TKIs can be considered for treatment of elderly CML patients. In our cohort of 60 elderly patients treated with nilotinib in first line, all patients at 3 months reached a CHR and 71.6% of them obtained early molecular response. At the last follow-up of 46.3 months, 84.4% reached sustained molecular response with 63.4% achieving a deep molecular response. These results are comparable to the results obtained in young patients both in the real world and in trials and it makes possible also in this setting of patients the possibility of TFR, as already suggested [5, 6]. However, age itself may not be the sole factor determining tolerability, as age is linked to comorbidities. A report presented at ASH 2010 by Khoury and colleagues [19] supports this data, by demonstrating that toxicities are linked to a higher comorbidity burden. In ENESTnd 10-year follow-up, cardiovascular side effects in the nilotinib arms were more frequent in elderly patients than in younger enrolled patients, although the percentage of elderly patients was significantly lower [16].

In our series, most patients reached good responses with very few side effects, although most of them (56/60) had mild/moderate CV comorbidities, in particular hypertension. In fact, apart from the hematological toxicity observed in some patients at the start of therapy, only 8 patients suffered of non-hematological side effects of low grade and only 6 of them showed cardiovascular events, just one severe. This low incidence of CV events may be due to a careful follow-up that we applied during treatment to monitor pre-existing comorbidities and avoid the onset of new ones, in anticipation of any side effects of TKIs. However, a potential limitation of this real-world experience is that the follow-up of the study is not long enough to observe the long-term rate of cardiovascular events which have been demonstrated increase with prolonged exposure.

Generally, in elderly patients, personalized selection of the most appropriate TKI is of paramount importance to guarantee long-term safety and compliance. When choosing second-generation TKI treatment, surveillance and optimal management of side effects are important to enable long-term continuous therapy. The incidence of side effects and the presence of comorbidities can be overcome by dose reduction, equally effective. In elderly patients, the dosage of the drug may be reduced once the desired response is achieved for that patient. Modified dosing schedules or reduced doses have been studied in elderly patients with CML [22], with the aim of maintaining the treatment response, enhancing overall tolerability, maintaining good medication adherence, and improving overall quality of life [23]. In our cohort, most patients started treatment at the standard dose (300 mg BID), and at the last follow-up, 12 patients (27.3%) out of 44 reduced nilotinib dose during treatment to avoid side effects, while 33 patients continued at the standard dose. Interestingly, one patient entered in TFR and he is in continuous deep MR after 2 years.

As referred also in < 65-year-old patients [23, 24], the dose reduction did not affect the effectiveness of the treatment; nevertheless, it impaired a possible TFR.

Although data on real-world use of first-, second-, and third-generation tyrosine kinase (TKI) inhibitors in chronic myeloid leukemia (CML) in the elderly are scarce, the analysis of available data suggests that such CML patients have an excellent prognosis and survival benefit, indistinguishable from that of younger patients [5, 25–30], as our data show.

Especially in older patients with a higher proportion of comorbidities, a more flexible dosing scheme may be warranted to increase tolerability while maintaining the deep molecular responses, also considering the possibility of TFR in this category of patients.

The use of second-generation TKIs in first line has shown a significantly higher proportion of patients to have optimal response at the 3-month milestone, which was recognized as an important prognostic factor and may increase the proportion of patients with deep molecular responses, who may potentially enter into treatment-free remission (TFR). Our data showed that in elderly patients with mild-moderate comorbidities, the treatment with second-generation TKIs might be used, possibly selecting the drug according to baseline comorbidities, to offer, especially in the category of patients aged between 65 and 75 years, the opportunity of a TFR, which, even if it requires regular monitoring, certainly guarantees a better quality of life.
